# Experimental and numerical investigation on a trimaran airwake, geometry modification

**DOI:** 10.1016/j.heliyon.2023.e21144

**Published:** 2023-10-20

**Authors:** Karim Akbari Vakilabadi, Hamid Reza Ghafari, Hassan Ghassemi

**Affiliations:** a Department of Mechanical Engineering, Imam Khomeini Naval University, Noshahr, Iran; b Marine and Hydrokinetic Energy Group, Department of Maritime Engineering, Amirkabir University of Technology, Tehran, Iran

**Keywords:** Trimaran, Airwake, Numerical simulation, Experimental measurement, PIV

## Abstract

The aerodynamic interaction between a helicopter and a trimaran ship's flight deck can be complex and have an impact on handling quality and performance, especially in turbulent conditions. This article presents research on the flight deck geometry of a trimaran vessel without the presence of a helicopter. Both Particle Image Velocimetry (PIV) and computational fluid dynamics (CFD) were used to analyze the effect of wind velocity on air pressure in the flight deck region. The study proposed and evaluated different geometries of the top structure at several air velocities to minimize pressure differences. The results of the numerical simulation were validated by experimental measurements using PIV, which showed that the effect of the Reynolds number on the non-dimensional pressure near the top structure is negligible except for the biggest Reynolds number (Re = 50e6), while at x/L = 0.5 the significant difference can be seen, however, the same result found for Re = 38e6 and 50e6. At the farthest distance (x/L = 1), the pressure difference for different Reynolds numbers case studies is negligible. Among the various geometries assessed, the maximum non-dimensional pressure differences along the lines show the highest value occurs for the base geometry (A) while geometries C and F show lower values, which have chamfering along the middle and side horizontal edges at a 45-degree angle and chamfering along all vertical and horizontal edges at a 30-degree angle.

## 1 Introduction

Ships are often used as mobile bases for helicopters, providing logistical support and transportation during military operations, search and rescue missions, and offshore oil and gas exploration. During helicopter operations, the helicopter's interactions with the ship's airwake can result in unpredictable and potentially hazardous flight conditions. Understanding the structure of a ship's airwake geometry and the aerodynamic interaction between a ship and a helicopter is crucial for ensuring safe and efficient helicopter operations from ships.

**Fig. 1:**
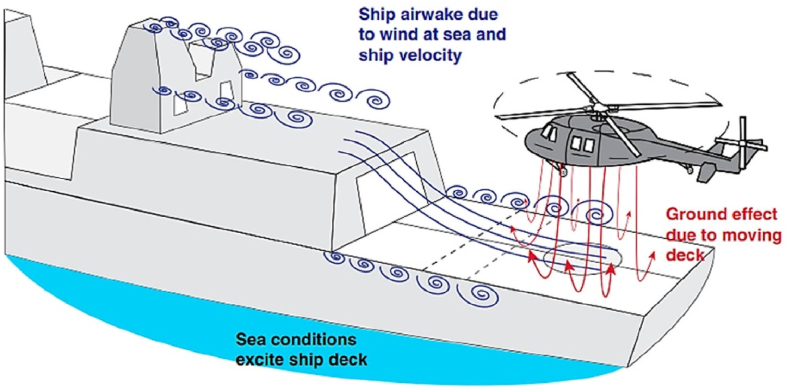
Schematic flow for Aerodynamic interaction between ship and helicopter in the airwake area [1].

The airwake geometry of a ship is influenced by several factors, including the ship's size, shape, speed, and direction of travel. The airwake behind a ship is characterized by a turbulent and complex flow field that includes vortices, eddies, and wakes. Fig. 1 shows the schematic flow for aerodynamic interaction between the ship and helicopter in the airwake area.

The airwake behind a ship can extend several hundred meters behind the vessel and can have a significant impact on the performance of helicopters operating in close proximity to the ship. When a helicopter approaches a ship for landing or takeoff, it enters the ship's airwake, which can result in significant changes to the helicopter's aerodynamic performance. The helicopter experiences increased turbulence and reduced lift due to the interaction with the ship's airwake. The helicopter's rotor blades may also experience unsteady and asymmetric loading, leading to instability and the potential for loss of control. The geometry of a ship's superstructure can have a significant impact on the shape and strength of the airwake that is applied to a helicopter. The presence of a tall superstructure on a ship can create a “wind shadow” behind the structure, which can result in a turbulent and unstable airwake for helicopters operating in close proximity to the ship. Additionally, the shape and location of radar antennas and other protruding structures on the superstructure can cause localized turbulence and vortices that can further complicate helicopter operations.

Ozgoren utilized a digital particle image velocimetry (DPIV) technique to analyze the downstream flow of circular, sharp-edged square, and 45-degree oriented square cylinders in uniform flow [2]. The analysis included the characterization of instantaneous vorticity, time-averaged velocity, and vorticity, root mean square (RMS) velocities, Reynolds stress correlations, and phase-averaged contours. Yagmur et al. used Large Eddy Simulation and PIV methods to compare the flow characteristics around an equilateral triangular cylinder [3]. Kilavuz et al. investigated an unmanned underwater vehicle near the free surface using PIV and numerical prediction [4]. They compared the 3-D and two-phase flow simulation generated using the Volume of Fluid (VOF) model with the Large Eddy Simulation (LES) turbulence model, with the PIV technique under the influence of the free surface.

Studies have indicated that the predominant frequency content of the unstable airwake is concentrated within the 0.2–2 Hz bandwidth [5]. This range coincides with the commonly accepted spectrum of frequencies for pilot closed-loop control. Previous research has characterized these frequencies by a cross-over frequency below 1.6 Hz [6]. Undertaking safety analysis for intricate missions is a challenging endeavor since it entails conducting hazardous and costly at-sea trials. Furthermore, to establish Ship-Helicopter Operational Limitations (SHOL), each ship-helicopter combination requires evaluation across a spectrum of wind speeds and directions [7]. In light of these complexities, developing a simulation tool for the helicopter-ship Dynamic Interface (DI) offers an effective solution to mitigate hazards and costs associated with prolonged at-sea testing campaigns [8]. By leveraging such a tool, safe landing trajectories can be identified, new flight control systems can be tested, and pilot training can be advanced via realistic simulation environments. Ultimately, the overarching aim of the NITROS project is to bolster rotorcraft operational safety, and the development of a simulation tool represents a significant step toward achieving this objective [9]. Lee and Zan measured the aerodynamic forces on a rotorless helicopter model in the ship airwake area experimentally in a large wind tunnel for a scale model of 1:50 [10]. The flight dynamics code incorporates ship airwake velocities through look-up tables, assuming that the ship airwake and rotor induced flow are superimposed. While this approach captures increased pilot workload due to the ship airwake's influence, it may not accurately represent cases where the helicopter is in close proximity to the ship's structure [5,11]. To assess the coupling effects on helicopter flight characteristics, Crozon et al. conducted four simulations: isolated ship, isolated rotor, shipborne rotor, and superposition of isolated rotor and ship. Both steady-state Reynolds-Averaged Navier-Stokes (RANS) with an actuator disk method and unsteady RANS using a blade-resolving representation of the rotor were investigated [12]. The results showed differences in rotor loading between isolated rotor in forward flight and near-deck operations, emphasizing the importance of coupling effects. This conclusion was further investigated to determine the minimum distance between the helicopter and a ground obstacle where the interaction can be considered negligible. The approximate distance was found to be 5 main rotor radii away from the obstacle, regardless of wind speed [13]. The study conducted by Taymourtash et al. focused on examining the interaction between a ship and a helicopter by employing a simplified geometry model. The aim was to assess the flow characteristics on the flight deck, and this was achieved through a series of experimental tests involving Particle Image Velocimetry surveys and Pressure measurements. These tests were carried out under varying wind conditions in the absence of a helicopter [14].

This paper introduces a new approach to studying the impact of top structure geometry on the air wake pattern of a trimaran vessel. The research focuses on determining the appropriate shape of the top structure by chamfering the horizontal and vertical edges of the back surface. To achieve this, both numerical and experimental studies were conducted using CFD and PIV methods to assess the airflow pattern that passes through the trimaran's top structure.

## 2 Methods

The technique of Particle Image Velocimetry enables flow field measurement in a non-destructive manner that does not disturb the current regime or its pattern. This general method allows for the measurement of the velocity field over a large area with high space and time accuracy. The main components required for PIV include a high-speed camera for image recording, a light-producing device, and light-reflecting particles that are introduced into the fluid. These particles should have a density similar to that of the fluid so they can move with the flow without affecting its pattern. Additionally, their size should be small enough to prevent any disturbance to the flow and large enough to reflect light for clear photography. A laser device is used as a light generator that illuminates the field using a field-mounted optical system, while a camera placed perpendicular to the screen captures images of the illuminated field. By analyzing successive images and particle tracking, the velocity vector of the field is determined. To avoid plagiarism, it is important to understand the information provided and present it in a way that is unique and original.

**Fig. 2:**
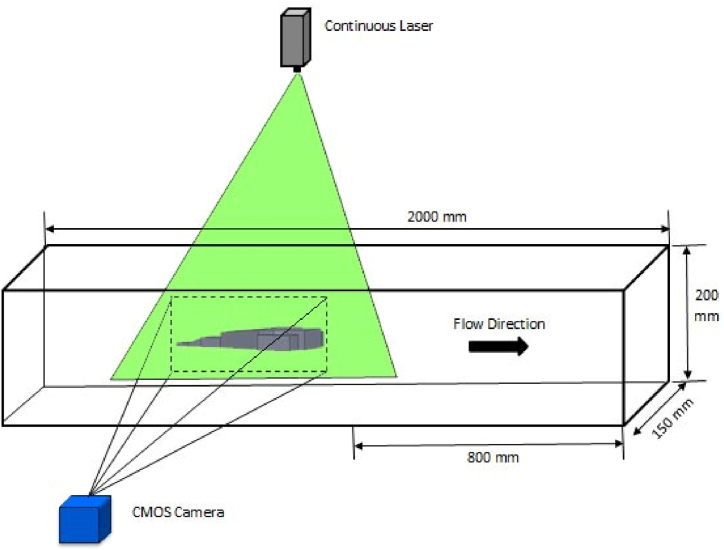
Schematic and dimensions of the experimental setup.

The PIV method used in this paper is shown in Fig. 2, which demonstrates the general principles of how it works. The current flows from left to right in the channel and after the particles pass the object, laser light is used to make them reflect, allowing the camera to take images and track the particles. By analyzing the time interval between two images and the displacement of the particle, velocity vectors can be determined. The laser used has a circular cross-section, and a small diameter, and is converted into a thin plate using cylindrical lens optics. It is a continuous laser with a wavelength of 532 nm and a power of 9 W. A CMOS-type PCO camera with a 1280 × 1024 pixel sensor is used to capture high-speed images. To produce particles of approximately 1 μm diameter, an ultrasonic mist producer is utilized. Image processing is carried out using MATLAB code.

## 3 Geometry and numerical setting

**Fig. 3:**
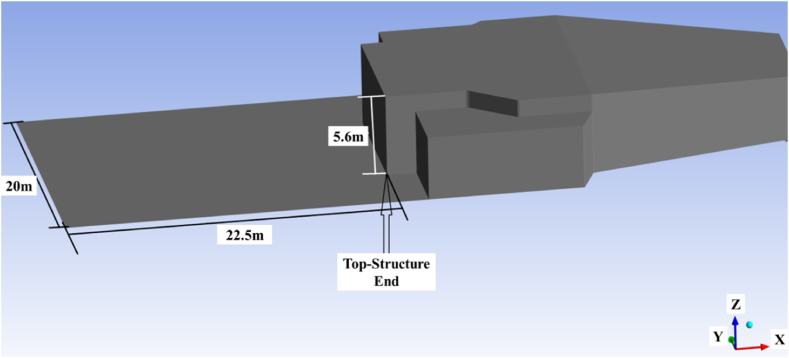
Geometry and the top-structure.

According to Fig. 3, the back sides are 22.5 m in length and 20 m in width. Additionally, the top structure reaches a height of 5.6 m. The Gambit software was utilized to model the geometry and computation domain, which were then imported into Fluent for simulation purposes. The simulation focused on analyzing the air flow surrounding the landing deck of the trimaran vessel in the presence of the top structure. Considering that the k-ε model, RNG has been able to predict well the shear stress on the walls, and considering the importance of the separation region, in this study, the model k-ε, RNG was chosen to simulate the turbulence model.

**Fig. 4:**
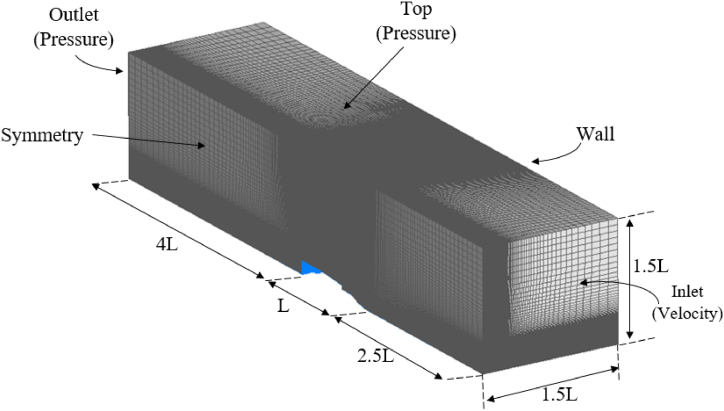
Computational domain, boundary conditions and meshing.

The computational domain was defined to have a length of 2.5L at the upstream end and 4L at the downstream end, with an additional 1.5L considered for its breadth, where L is the top structure length. The meshing, boundary conditions, and computational domain can be observed in Fig. 4. A structured mesh was designed for the domain at an appropriate distance from the walls. The inlet surface was examined for velocity considerations, while the outlet surface was analyzed for pressure measurements. To reduce computational time, the simulation accounted for the symmetric geometry of the landing deck structure and the top structure, thus necessitating the consideration of the symmetry condition.

**Fig. 5:**
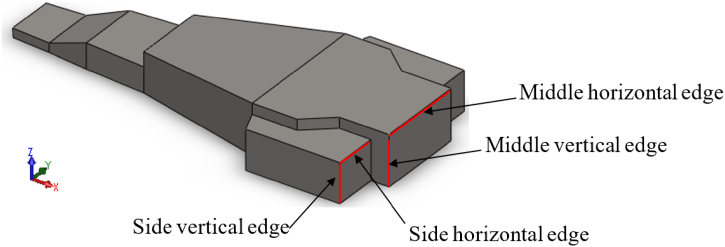
Base geometry and its important edges for top structure modification.

In an effort to reduce pressure differences in the wake area, modifications were made to the top-structure geometry which involved adding chamfering angles to both horizontal and vertical edges. The key edges that were altered as part of this process are depicted alongside the original base geometry in Fig. 5.

**Fig. 6:**
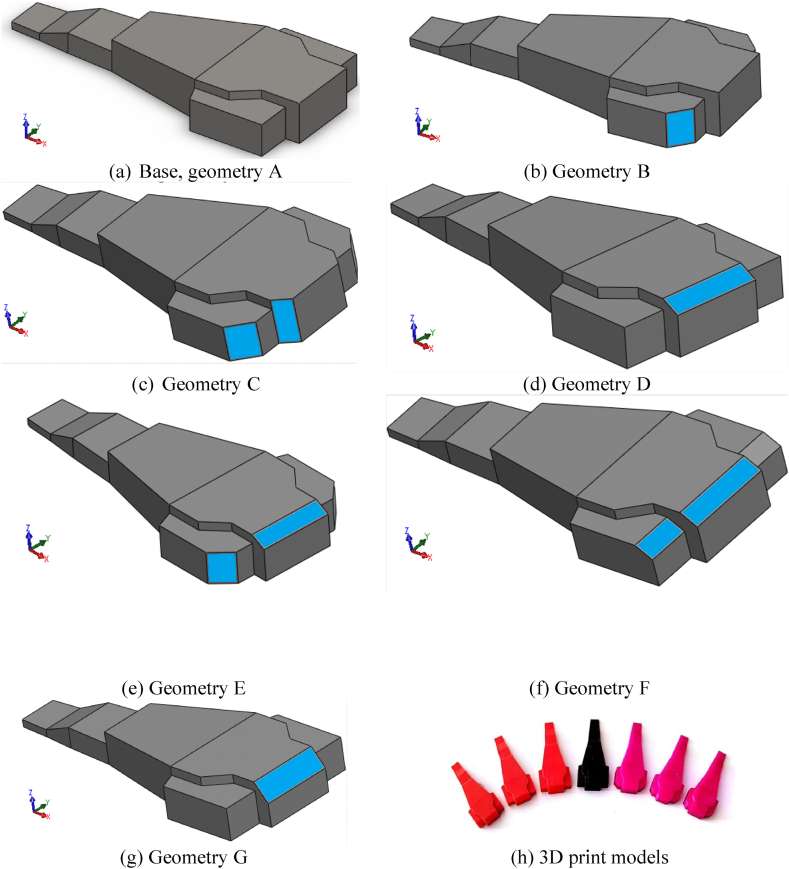
Different case study of top structure.

Using the base geometry of the top structure as a starting point, six alternative geometries were proposed and listed in Table 2 and Fig. 6. These geometries feature chamfered horizontal and vertical edges (as illustrated in Fig. 5) with the specific angle of chamfering noted in Table 1.

**Table 1:** Top-structure case study and description.

Case study	Description of Geometry	Chamfer angle (Degree)
A	Sharp edge at all vertical and horizontal edges (Base)	–
B	Chamfering along the side horizontal edge	45
C	Chamfering along middle and side horizontal edges	45
D	Chamfering along middle vertical side	30
E	Chamfering along middle and side vertical edges	30
F	Chamfering along all vertical and horizontal edges	30
G	Chamfering along middle vertical edge	50

**Table 2:** Maximum pressure difference on different levels in mid-section.

	$\left(Pmax-Pmin\right)\;/P_{dyn\;0}$			
Reynolds Number	Re = 12e6	Re = 25e6	Re = 38e6	Re = 50e6
Line 1 (Z = 0.0h)	0.37	0.64	0.55	0.49
Line 2 (Z = 0.33h)	0.36	0.63	0.55	0.50
Line 3 (Z = 0.66h)	0.46	0.56	0.49	0.47
Line 4 (Z = 1.0h)	0.48	0.43	0.41	0.50

## 4 Mesh study and validation

### 4.1 Mesh study

**Fig. 7:**
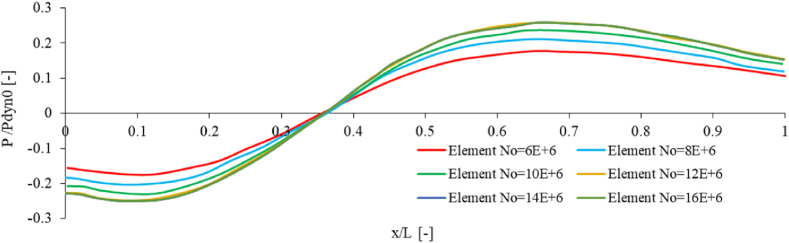
Effect of element number on the non-dimensional pressure along the line (Z = 0.66h).

As part of this investigation, a mesh study was undertaken to verify the independence of numerical results from the number of elements used. Specifically, the study examined the base geometry under a wind velocity of 40 m/s and Re = 50e6. Results were collected at distances of 0.66h from the landing deck for different element numbers, as visualized in Fig. 7. Equation $P/P_{dyn\;0}$ is used for the non-dimensional pressure in which $P_{dyn\;0}=\frac{1}{2}\rho V_{0}^{2}$ and $V_{0}$ is the upstream velocity.

The numerical results indicate that there is a substantial variation between the outcomes when the number of elements increases from 6.0e6 to 12e6. However, as the number of elements further increases, the difference in results becomes insignificant. Thus, for the numerical simulation, 12e6 elements were chosen as they seem appropriate. The numerical simulation was carried out using 7 computing cores, 2.2 GHz processors, and 16 GB of shared memory. It took 24 h to compute the numerical simulation with turbulent flow.

### 4.2 Validation

**Fig. .8:**
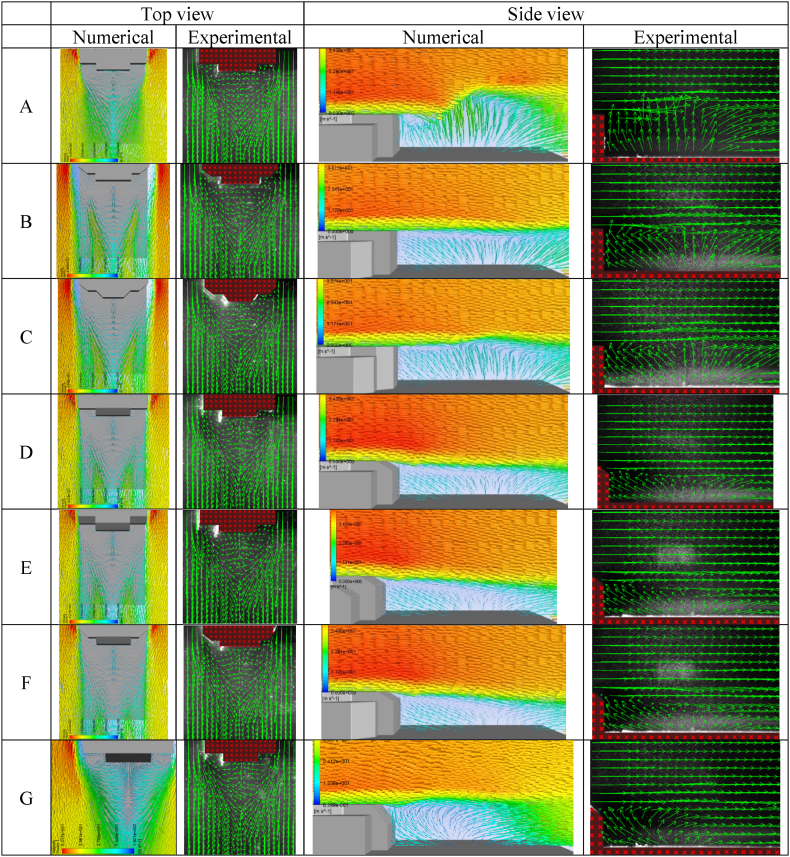
Validation via velocity vectors, numerical simulation verses experimental measurements using PIV method, geometries A-G.(Re = 50e6).

The accuracy of the numerical simulation of the airflow field over the top structure of a trimaran vessel was confirmed through a comparison of the CFD analysis results with experimental measurements obtained using the PIV method. To ensure the reliability of the experimental data, the study was conducted twice on two vertical planes aligned with the fluid's free flow for each of the seven different geometries. Overall, 14 tests were performed, and the repeatability of each experiment was analyzed. Fig. 8 depicts a comparison between the velocity field vector snapshots obtained from the experimental images and numerical simulations.

**Fig. 9:**
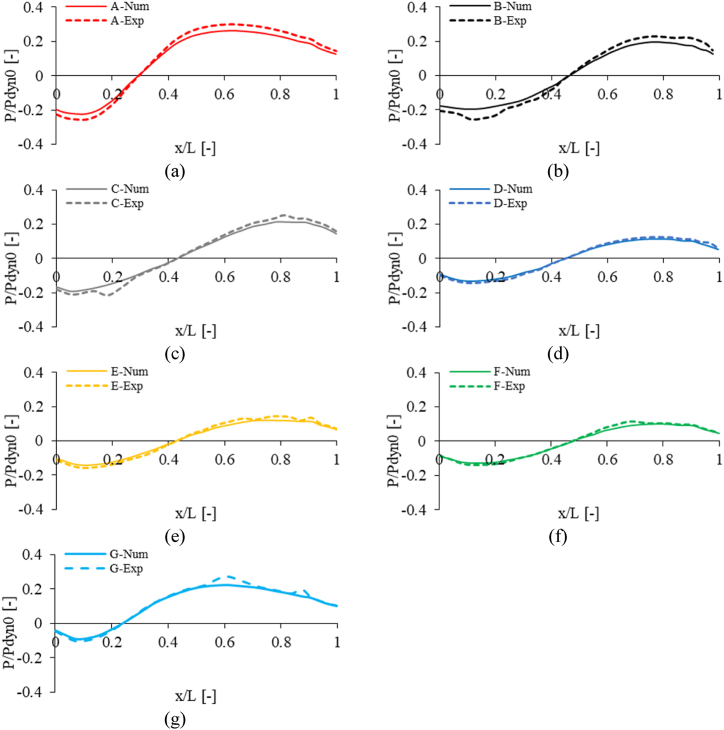
Comparison of non-dimensional pressure between CFD and PIV method (Re = 50e6).

**Fig. 10:**
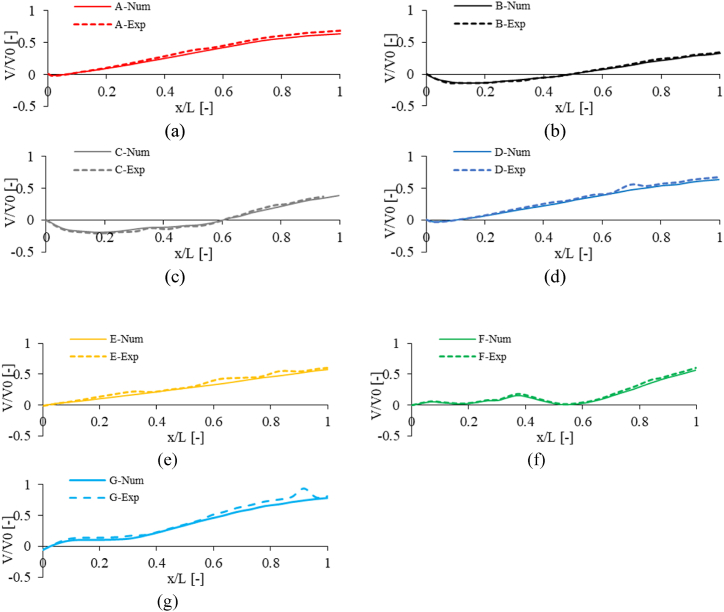
Comparison of non-dimensional velocity between CFD and PIV method (Re = 50e6).

Fig. 9, Fig. 10 compare the non-dimensional pressure and non-dimensional velocity between CFD and PIV methods at Re = 50e6 for seven geometries. The Results were obtained in the wind direction for a line at the mid-section plane with Z = 2.8 m from the landing deck. The numerical results highlight the good agreement with the experimental measurement.

## 5 Results and discussion

**Fig. 11:**
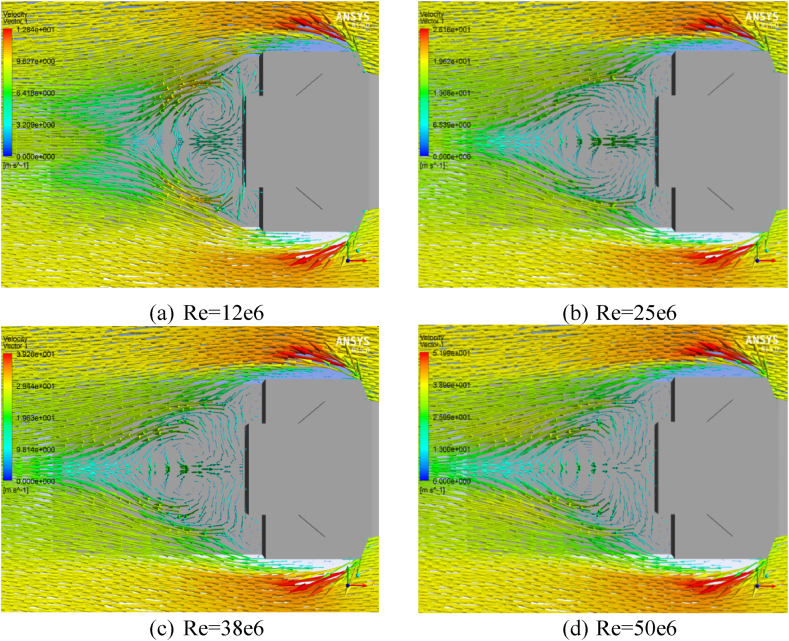
Velocity vectors on X–Y plane at Z = 2.8 m from the landing deck.

**Fig. 12:**
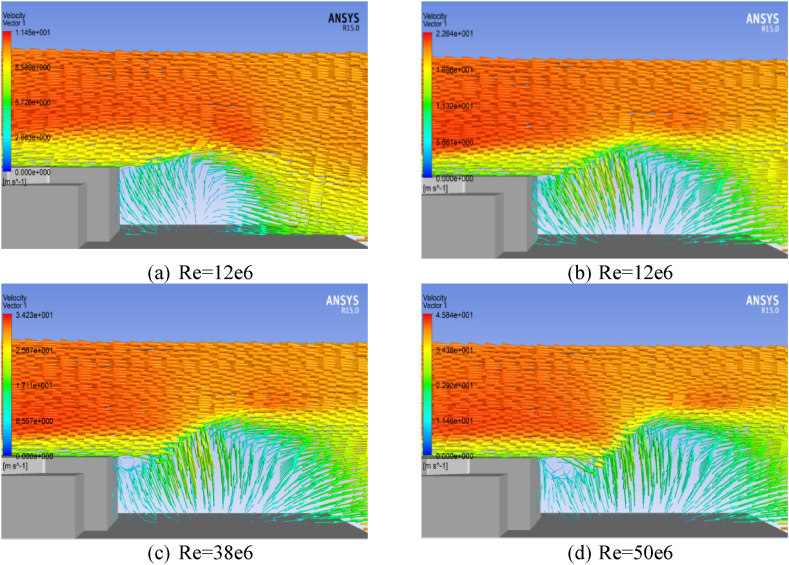
Velocity vectors in X-Z symmetry plane, base geometry.

Numerical analysis was performed to investigate the ship Airwake of a trimaran vessel with a base geometry on the landing deck, under varying Reynolds Numbers 12e6, 25e6, 38e6, and 50e6. In Fig. 11, Fig. 12 velocity vectors at different wind velocities are displayed on the X–Y plane, positioned 0.66h away from the landing deck (Z = 2.8 m) and on the Z-X symmetry plane, respectively.

Fig. 11, Fig. 12 indicate that the Reynolds number of 50e6 resulted in the most critical scenario, generating a significant vortex behind the top structure.

**Fig. 13:**
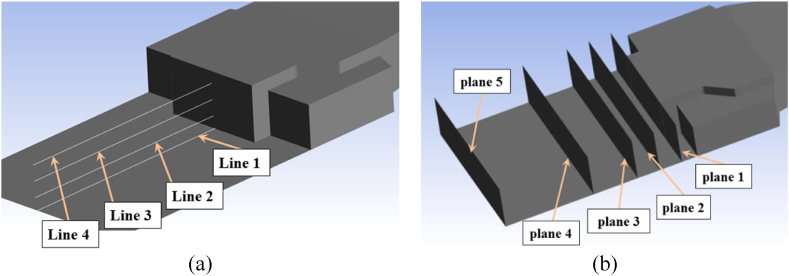
a) Four lines at intervals of 0, 0.33h, 0.66h and h from the landing deck, b) Five planes at intervals 0, 0.125L, 0.25L, 0.5L and L behind the top-structure.

To illustrate the pressure fluctuations behind the top-structure, Fig. 13a depicts four lines positioned at intervals of 0, 0.33h, 0.66h, and h(4.4 m) from the landing deck, as well as five planes with distances of 0, 0.125L, 0.25L, 0.5L, and L from top structure (Fig. 13b), where L is 22.5 m.

**Fig. 14:**
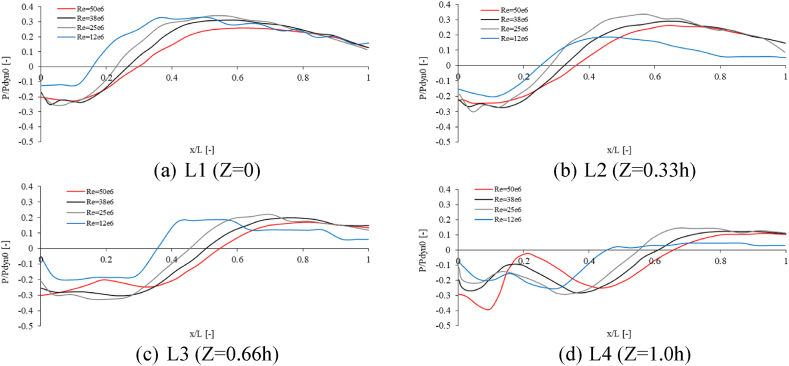
Non-dimensional pressure changes along different lines.

Fig. 14 displays the non-dimensional pressure changes along four distinct lines, as illustrated in Fig. 13a.

The results show an upward trend in pressure with increasing distance from the back surface of the top structure. Additionally, local peaks and troughs are observed at some locations. As the distance from the landing deck increases, the zero pressure point occurs further from the back surface of the top structure. For Re = 50e6 result shows that on the plane of the landing deck (z = 0), the pressure is zero at a distance of 0.3L, while on the highest line (z = 1h), the zero pressure point occurs at a distance of 0.65L. The highest pressure at higher levels from the landing deck also occurs far from the back surface of the top structure.

Table 2 reveals the maximum pressure variance along each line for different Reynolds numbers. Based on the results, the maximum pressure difference at a higher level from the landing deck occurred at a greater Reynolds number (Re = 50e6) while the bigger difference belongs to Re = 25e6 for Z = 0, Z = 0.33h, and Z = 0.66h.

**Fig. 15:**
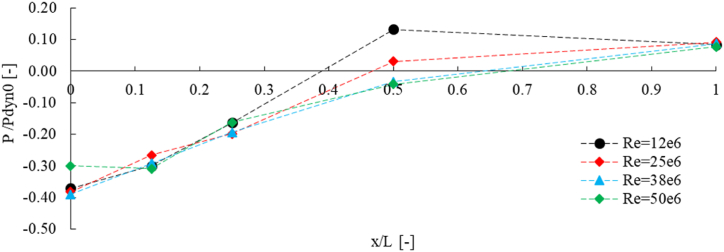
Comparison of the average non-dimensional pressure at five planes between different Reynolds numbers.

In Fig. 15, the average pressure at different Reynolds numbers is compared. Results obtained from measurements at five different planes as shown in Fig. 13a. The results indicate that at different Reynolds numbers, the non-dimensional pressure near the top structure is negligible except for greater Reynolds number (Re = 50e6), while at x/L = 0.5 the significant difference can be seen, however, the same result found for Re = 38e6 and 50e6. At the farthest distance(x/L = 1), the pressure difference for different Reynolds numbers case studies is negligible.

**Fig. 16:**
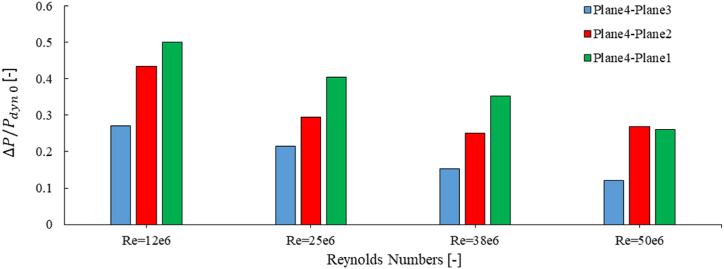
The average pressure difference between plane 4 and planes 1 to 3.

The average pressure was measured on five planes with distances of 0, 0.125L, 0.25L, 0.5L, and L from top structure, where L is 22.5 m. The differences between these pressures are shown in Fig. 16.

The results obtained for the base geometry show that the as Reynolds number increases, the pressure difference decreases. Besides it found that the difference between planes 4 and 1 is more than other planes. However, at bigger Reynolds numbers (Re = 50e6), the difference between Plane4-Plane1 is near than Plane4-Plane2. A comparison of the Reynolds number shows that the minimum pressure difference belongs to Re = 50e6.

The proposed case studies (Table 2) were investigated at Re = 50e6. Table 3 lists the maximum non-dimensional pressure differences along the lines. Results were obtained at Reynolds numbers of 50e6. As can be seen, the highest value occurs for the base geometry (A) while geometries C and F show lower values.

**Table 3:**
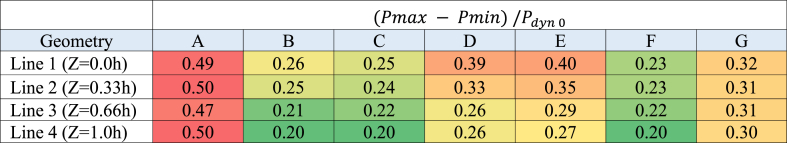
Comparison of maximum difference for non-dimensional pressure between different geometry.

The difference in average non-dimensional pressure between plane 4 and planes 1, 2, and 3 are listed in Table 4 for different case studies at Re = 50e6. In general, the difference between Plane 4 and Plane 3 is the lowest for all cases. The maximum value belongs to geometries A and G while the minimum occurs for geometry C and F.

**Table 4:**
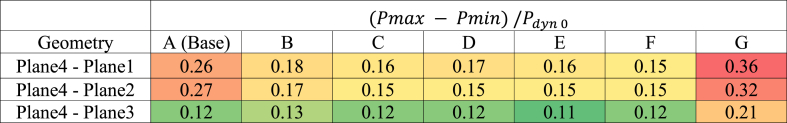
The difference in average non dimensional pressure between plane 4 and planes 1, 2 and 3 (Re = 50e6).

Given the fact that the helicopter is almost centered on Plane 4 and faces almost in line with Plane 2, the best criterion is to check the difference in average pressure between Plane 4 and Plane 2. Therefore, it can be concluded that the lowest maximum pressure difference belongs to geometries C and F, which have chamfering along the middle and side horizontal edges at a 45-degree angle and chamfering along all vertical and horizontal edges at a 30-degree angle, respectively. These geometries can be recommended as having the best performance compared to all case studies.

## 6 Conclusions

In this paper, the effect of top structure geometry on the air wake pattern of a trimaran vessel was investigated to obtain the proper geometry of the top structure by chamfering the horizontal and vertical edges of the top structure's back surface. Numerical and experimental studies were carried out using CFD and PIV methods, respectively, to evaluate the airflow pattern that passes through the top structure of a trimaran. The pressure at different levels from the landing deck and different distances from the top structure in the horizontal direction was analyzed. The numerical analysis of the ship airwake for the landing deck of a trimaran vessel with base geometry was carried out at different Reynolds numbers of 12e6, 25e6, 38e6 and 50e6 and compared with experimental measurements using PIV methods. The most important conclusions of the present study are as follows:

1 The most critical condition occurred at a Re = 50e6, which generated the greatest vortex behind the top structure:
2 Although local peaks and troughs are observed for non-dimensional pressure, an upward trend is obtained with increasing distance from the back surface of the top structure.
3 As the distance from the landing deck increases, the zero pressure occurs at a further distance from the back surface of the top structure.
4 On the plane of the landing deck (z = 0), zero pressure can be found at a distance of 0.3L from the top structure, and on the highest plane (z = 4.4 m), the zero pressure value has occurred at a distance of 0.66L meters.
5 The highest pressure at higher levels from the landing deck has also occurred at distances far from the back surface of the top structure.
6 The effect of Re on the non-dimensional pressure near the top structure is negligible except for the biggest Reynolds number (Re = 50e6), while at x/L = 0.5 the significant difference can be seen, however, the same result found for Re = 38e6 and 50e6. At the farthest distance(x/L = 1), the pressure difference for different Reynolds numbers case studies is negligible.
7 The maximum non-dimensional pressure differences along the lines show the highest value occurs for the base geometry (A) while geometries C and F show lower values, which have chamfering along the middle and side horizontal edges at a 45-degree angle and chamfering along all vertical and horizontal edges at a 30-degree angle.

## Data availability statement

The authors confirm that the data supporting the findings of this study are available within the article and its Supplementary material. Raw data that support the findings of this study are available from the corresponding author, upon reasonable request.

## CRediT authorship contribution statement

**Karim Akbari Vakilabadi:** Conceptualization, Formal analysis, Methodology, Supervision, Writing – original draft, Writing – review & editing. **Hamid Reza Ghafari:** Conceptualization, Formal analysis, Methodology, Validation, Writing – original draft. **Hassan Ghassemi:** Conceptualization, Methodology, Supervision, Validation, Writing – original draft, Writing – review & editing.

## Declaration of competing interest

The authors declare that they have no known competing financial interests or personal relationships that could have appeared to influence the work reported in this paper.
